# RORγt-dependent antigen-presenting cells direct regulatory T cell-mediated tolerance to food antigen

**DOI:** 10.1101/2024.07.23.604803

**Published:** 2024-07-25

**Authors:** Liuhui Fu, Rabi Upadhyay, Maria Pokrovskii, Gabriela Romero-Meza, Adam Griesemer, Dan R. Littman

**Affiliations:** 1 Department of Cell Biology, New York University School of Medicine, New York, NY, USA.; 2 Perlmutter Cancer Center, NYU Langone Health, New York, NY, USA.; 3 Calico Life Sciences, LLC, South San Francisco, CA, USA.; 4 NYU Langone Transplant Institute, NYU Langone Health, New York, NY, USA.; 5 Howard Hughes Medical Institute, New York, NY, USA.

## Abstract

The gastrointestinal tract is continuously exposed to foreign antigens in food and commensal microbes with potential to induce adaptive immune responses. Peripherally induced T regulatory (pTreg) cells are essential for mitigating inflammatory responses to these agents^1-4^. While RORγt^+^ antigen-presenting cells (RORγt-APCs) were shown to program gut microbiota-specific pTregs^5-7^, understanding of their characteristics remains incomplete, and the APC subset responsible for food tolerance has remained elusive. Here, we demonstrate that RORγt-APCs are similarly required for differentiation of food antigen-specific pTregs and establishment of oral tolerance. The ability of these cells to direct both food and microbiota-specific pTreg cell differentiation is contingent on expression of RORγt and on a unique cis-regulatory element within the *Rorc* gene locus (*Rorc(t)* +7kb). Absent this +7kb element, there was a notable increase in food antigen-specific T helper 2 (Th2) cells in lieu of pTregs, leading to compromised tolerance in a mouse asthma model. By employing single-cell analyses across these models, as well as freshly resected mesenteric lymph nodes from a human organ donor, we identified a rare subset of evolutionarily conserved APCs that are dependent on RORγt, uniquely express the Prdm16 transcription factor, and are endowed with essential mediators for inducing pTreg cell differentiation. Our findings suggest that a better understanding of how RORγt-APCs develop and how they regulate T cell responses to food and microbial antigens could offer new insights into developing therapeutic strategies for autoimmune and allergic diseases as well as organ transplant tolerance.

Antigen-presenting cells (APCs) play pivotal roles in orchestrating immune responses. They direct T cell outcomes, ranging from diverse effector to suppressive programs, according to potential threats from pathogenic microbes and the environmental context^8,9^. The adaptive immune response has evolved to be tolerant not only to self-antigens, but also to suppress responses to antigens present in the diet and in the mutualistic microbiota^10-12^. Tolerance to at least some commensal microbes with the potential to induce inflammation is mediated by pTregs that are programmed by recently-discovered RORγt-APCs^5-7^. Although these APCs express CD11c and Zbtb46, canonical markers of dendritic cells, they appear to have distinct features that are not yet fully understood. Which APCs serve a similar function in directing the differentiation of pTregs that mediate tolerance to oral antigens in the proximal alimentary tract remains uncertain. Classical dendritic cells were proposed to be inducers of dietary antigen-specific pTregs, and it was also suggested that there is redundancy for this function among different types of APCs^13,14^. In this study, we set out to determine if the nuclear receptor RORγt, known to have critical roles in thymopoiesis, peripheral T cell differentiation, and development of innate lymphoid cells, is similarly required for the development and/or function of the newly described APCs. We identified a unique evolutionarily conserved pTreg-inducing cell subset whose development is contingent on RORγt expression and in whose absence there was loss of tolerance to both oral antigens and intestinal microbiota. Our results suggest that these APCs have critical roles in immune homeostasis and that their dysfunction would contribute to inflammatory disease.

## Tolerogenic APC function requires *RORγt*

Tolerogenic RORγt-APCs have been proposed to be MHCII^+^ type 3 innate lymphoid cells (ILC3) or subsets of recently described Janus cells (JC) and Thetis cells (TC), some of which express Aire^5-7^. Notably, targeting by RORγt-cre of conditional alleles for MHCII, α_v_β_8_ integrin, and CCR7 in these APCs resulted in loss of pTregs, indicating that the APCs express RORγt during ontogeny, but it remained uncertain whether RORγt is itself required for these cells to develop or perform their function. We previously showed that inactivation of the same target genes in CD11c-cre mice also resulted in loss of pTreg-inducing APC function.

**Extended Data Fig. 1 ∣:**
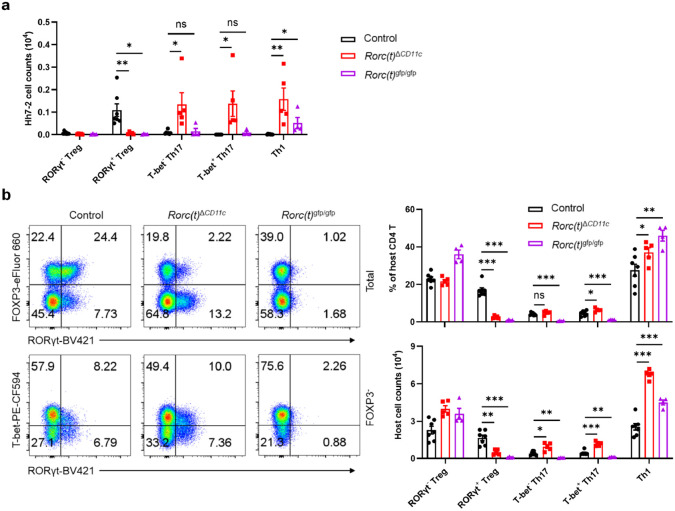
RORγt expression in CD11c lineage APCs is necessary for directing the differentiation of gut microbiota-specific pTregs. **a**, Numbers of *Hh*-specific pTreg, Th17 and Th1 cells in the LILP of *Hh*-colonized control (n = 7) , *Rorc(t)*^Δ*CD11c*^ (n = 5) and *Rorc(t)*^gfp/gfp^ (n = 4) mice at 14 days after adoptive transfer of naïve Hh7-2tg CD4^+^ T cells. **b**, Phenotype of host CD4 T cells in the LILP of mice shown in (**a**), with representative flow cytometry profiles (left) and aggregate quantitative data (right). The lower panels of flow cytometry plots are gated on FOXP3^−^ cells. Data are pooled from two independent experiments. Data are means ± s.e.m.; ns, not significant; statistics were calculated by unpaired two-sided t-test.

**Fig. 1 ∣:**
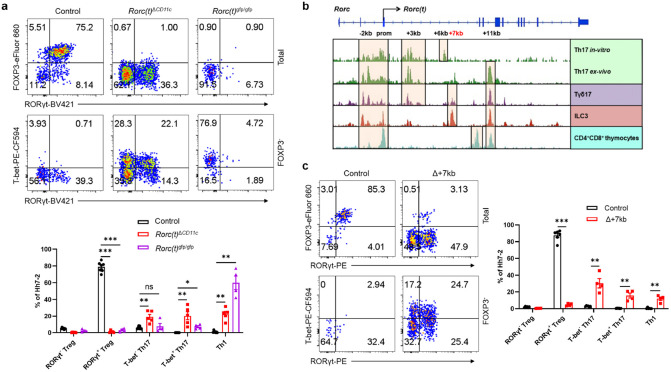
RORγt is required by tolerogenic APCs to promote microbiota-specific pTreg differentiation. **a**, Representative flow cytometry plots (top) and frequencies (bottom) of *Hh*-specific pTreg (FOXP3+RORγt+^-^), Th17 (FOXP3^−^RORγt+T-bet^+/−^) and Th1 (FOXP3^−^RORγt^−^T-bet^+^) cells in the LILP of *Hh*-colonized control (*Rorc(t)*^fl/gfp^, *Rorc(t)*^+/gfp^, and *Cd11c*^cre^*Rorc(t)*^+/gfp^; n = 7), *Rorc(t)*^Δ*CD11c*^ (*Cd11c*^cre^*Rorc(t)*^fl/gfp^; n = 5) and *Rorc(t)*^gfp/gfp^ (n = 4) mice at 14 days after adoptive transfer of naïve Hh7-2tg CD4+ T cells. The lower panels of flow cytometry plots are gated on FOXP3^−^cells. **b**, Bulk ATAC-seq data showing accessible regions in the *Rorc* locus of several RORγt-expressing cell types, including CD4^+^CD8^+^ thymocytes, *in vitro* differentiated Th17 cells, and SILP-derived Th17 (TCRβ^+^CD4^+^IL23R-GFP^+^) cells, Tγδ17 (TCRγδ^+^IL23R-GFP^+^) cells and ILC3 (Lin^−^IL-7R^+^Klrb1b^+^NK1.1^−^). **c**, Phenotype of *Hh*-specific T cells in the LILP of *Hh*-colonized control (*Rorc(t)* +7kb^+/+^; n = 6) and Δ+7kb (*Rorc(t)* +7kb^−/−^; n = 4) mice at 14 days after adoptive transfer of naïve Hh7-2tg CD4^+^ T cells. The lower panels of flow cytometry plots are gated on FOXP3^−^ cells. Data in **a** are pooled from two independent experiments. Data in **c** are representative of two independent experiments. Data are means ± s.e.m.; ns, not significant; statistics were calculated by unpaired two-sided t-test.

To investigate a potential role for RORγt in these APCs, we therefore inactivated *Rorc(t)* in CD11c-cre mice and determined the fate of T cells specific for the large intestine pathobiont *Helicobacter hepaticus (Hh)*. Naïve *Hh*-specific CD4^+^ T cells from Hh7-2 TCR transgenic mice^15^ were transferred into *Hh*-colonized mice. Two weeks post-transfer, the large intestine lamina propria (LILP) of control mice displayed a predominance of pTregs expressing both RORγt and FOXP3 (Fig. 1a and Extended Data Fig. 1a). In contrast, in the LILP of *Cd11c*^cre^*Rorc(t)*^fl/gfp^ (*Rorc(t)*^Δ*CD11c*^) mice, the differentiation of adoptively transferred *Hh*-specific pTregs was abrogated. Instead, these mice exhibited an increase in RORγt- and T-bet-expressing *Hh*-specific T cells (Fig. 1a and Extended Data Fig. 1a), indicative of a shift towards a pro-inflammatory profile. Similarly, among endogenous T cells in these mutant mice there were fewer RORγt^+^ pTregs and substantially more inflammatory T helper 17 (Th17)/Th1 cells (Extended Data Fig. 1b). Consistent with findings in *Rorc(t)*^Δ*CD11c*^ mice, *Hh*-specific T cells in *Rorc(t)*^gfp/gfp^ mice failed to differentiate into pTregs, and instead adopted a Th1 phenotype, expressing T-bet (Fig. 1a and Extended Data Fig. 1a,b). These results indicate that RORγt expression in CD11c lineage APCs is required for them to direct gut microbiota-specific pTreg cell differentiation.

## Lineage-specific *Rorc(t) cis* elements

**Extended Data Fig. 2 ∣:**
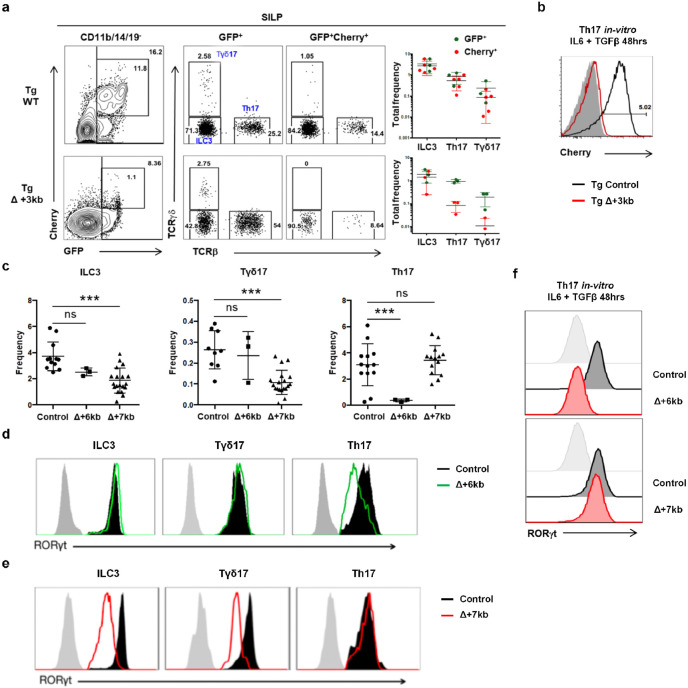
Characterization of lineage-specific *Rorc(t)* cis-regulatory elements. **a**,**b**, Representative flow cytometry plots and summary graphs depicting comparison of SILP GFP^+^ and mCherry^+^ populations (**a**) as well as mCherry expression of *in vitro* differentiated Th17 cells (**b**) in BAC transgenic mice bred to RORγt GFP reporter mice (heterozygous for gfp knockout allele), Tg (Control *Rorc(t)*-mCherry);*Rorc(t)*^+/gfp^ and Tg (Δ+3kb *Rorc(t)*-mCherry);*Rorc(t)*^+/gfp^ mice. **c**, Summary of indicated SILP RORγt^+^ populations as frequency of SILP mononuclear cells from control, Δ+6kb and Δ+7kb mice. **d**,**e**, Representative histograms showing RORγt expression in the SILP RORγt^+^ populations from mice shown in (**c**). **f**, RORγt expression among *in vitro* differentiated Th17 cells isolated from mice shown in (**c**). Data are means ± s.d.; ns, not significant; statistics were calculated by unpaired two-sided t-test.

RORγt is a transcription factor whose expression is largely confined to diverse lymphoid lineage cells, in which it contributes to distinct phenotypic programs^16-21^. Because different cis-regulatory elements (CREs) within a gene locus can govern its cell-specific expression, as best exemplified by the erythroid-specific enhancer in *Bcl11a*^22^, a therapeutic target for sickle cell disease, we hypothesized that distinct CREs within the *Rorc* locus may selectively modulate expression in the RORγt^+^ lineages, including the pTreg-inducing APCs. While prior research delineated several CREs involved in RORγt expression in Th17 cells and ILC3, *Rorc* regulatory regions in other cell types that express the transcription factor remain less characterized^23-25^. To identify cell type-specific regulatory sequences, we conducted bulk ATAC-seq analyses in several RORγt-expressing cell types, including CD4^+^CD8^+^ thymocytes, *in vitro* differentiated Th17 cells, and small intestine lamina propria (SILP)-derived Th17 cells, Tγδ17 cells, and presumptive type 3 innate lymphoid cells (ILC3). These studies revealed distinct patterns of chromatin accessibility within the *Rorc* locus across the different cell types (Fig. 1b). Notably, regions situated +6kb and +7kb from the *Rorc(t)* transcription start site exhibited pronounced accessibility in *in vitro* differentiated Th17 cells and intestinal ILC3, respectively (Fig. 1b). Additionally, the +11kb element exhibited open chromatin configuration in all RORγt^+^ cell types, with the notable exception of *in vitro* polarized Th17 cells (Fig. 1b), consistent with our previous findings^24^. Further studies using dual reporter BAC transgenic mice specifically lacking a +3kb element (Tg (Δ+3kb *Rorc(t)*-mCherry);*Rorc(t)*^+/gfp^) indicated that *Rorc(t)* +3kb is a pivotal enhancer in Th17 and Tγδ17 cells *in vivo*, as well as *in vitro* differentiated Th17 cells, but not in ILC3 (Extended Data Fig. 2a,b).

To further explore the functional importance of the *Rorc(t)* +6 kb and +7kb elements, we engineered mice with deletions of these sequences. *Rorc(t)* +6kb^−/−^ (Δ+6kb) mice had significant reduction in both the proportion of Th17 cells in the SILP and level of RORγt expression within the remaining cells, but no change in ILC3 and Tγδ17 cells (Extended Data Fig. 2c,d). Notably, when naïve CD4 T cells from these knockout mice were subjected to Th17 differentiation conditions, they failed to upregulate RORγt (Extended Data Fig. 2f), confirming the regulatory importance of the +6kb region. In contrast, analysis of *Rorc(t)* +7kb^−/−^ (Δ+7kb) mice showed significantly reduced RORγt^+^ SILP ILC3 and Tγδ17 populations, with reduced RORγt expression in the remaining cells, but no effect in Th17 cells (Extended Data Fig. 2c,e). Naïve CD4^+^ T cells isolated from Δ+7kb mice displayed normal RORγt expression upon *in vitro* Th17 cell differentiation (Extended Data Fig. 2f), consistent with a role of this element only in innate-type lymphocytes.

**Extended Data Fig. 3 ∣:**
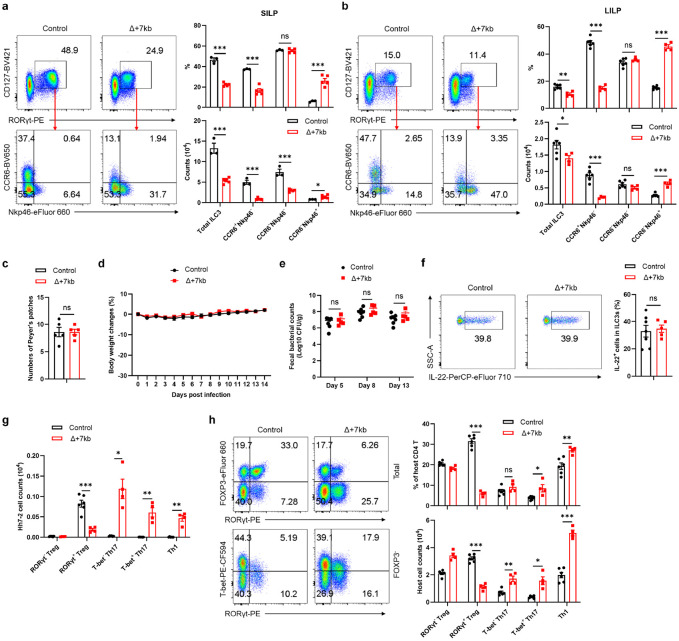
Role of *Rorc(t)* +7kb in ILC3 development and function. **a**,**b**, Phenotype of ILC3 (CD45^+^Lin^−^CD127^+^RORγt^+^) subsets in the SILP (**a**) and LILP (**b**) of control and Δ+7kb mice. SILP: control mice, n = 3; Δ+7kb mice, n = 5. LILP: control mice, n = 6; Δ+7kb mice, n = 4. **c**, Numbers of Peyer’s patches in control (n = 5) and Δ+7kb (n = 5) mice. **d**-**f**, Body weight changes (**d**), fecal *C. rodentium* counts (**e**) and frequencies of IL-22^+^ cells in RORγt^+^ ILC3 (day 14, *ex vivo* stimulation with IL-23) in the LILP (**f**) of control (n = 7) and Δ+7kb (n = 5) mice post *C. rodentium* infection. **g**, Numbers of *Hh*-specific T cells in the LILP of *Hh*-colonized control (n = 6) and Δ+7kb (n = 4) mice at 14 days after adoptive transfer of naïve Hh7-2tg CD4^+^ T cells. **h**, Phenotype of host CD4 T cells in the LILP of mice shown in (**g**). The lower panels of flow cytometry plots are gated on FOXP3^−^ cells. Data in **d**-**f** are pooled from two independent experiments. Data in **a**-**c**,**g**,**h** are representative of two (**a**,**b**,**g**,**h**) or three (**c**) independent experiments. Data are means ± s.e.m.; ns, not significant; statistics were calculated by unpaired two-sided t-test.

The ILC3 subsets in the SILP and LILP of mutant mice were skewed towards Nkp46-expressing NCR^+^ ILC3, with reduction in CCR6-expressing LTi-like ILC3 (Extended Data Fig. 3a,b). Notably, the number of Peyer's patches remained unchanged (Extended Data Fig. 3c), indicating that RORγt-dependent lymphoid tissue inducer (LTi) cell functionality was not compromised. When the Δ+7kb mice were challenged with the enteric pathogen *Citrobacter rodentium*, there was no difference in bacterial titers and weight loss compared to wild-type controls, nor was there any defect in IL-22 production (Extended Data Fig. 3d-f), which is essential for bacterial clearance^26^. These results suggested that mature ILC3 in the LILP of Δ+7kb mice remain functional, despite the reduction or loss of RORγt, which is required early for ILC3 development and for repression of T-bet, which, in turn, promotes expression of Nkp46 and transition to the ILC1 phenotype. This finding is consistent with previous studies showing that while RORγt is crucial for restraining transcriptional networks associated with type 1 immunity, it is not essential for robust IL-22 production among mature ILC3^27,28^.

## Tolerogenic APC-specific *Rorc(t)* CRE

Despite the apparent maintenance of ILC3 function in the Δ+7kb mice, there was severe disruption of adoptively transferred *Hh*-specific pTreg cell differentiation in the LILP and expansion of inflammatory Th17/Th1 cells (Fig. 1c and Extended Data Fig. 3g). There was also a reduction of endogenous RORγt^+^ pTregs and increase of Th17/Th1 cells in the *Hh*-colonized mice (Extended Data Fig. 3h). Thus, the *Rorc(t)* +7kb CRE is required for RORγt-APCs to promote microbiota-specific pTreg cell differentiation even though known ILC3/LTi cell functions remain intact, which raise the possibility that such APCs belong to a different cell lineage than the ILCs.

**Extended Data Fig. 4 ∣:**
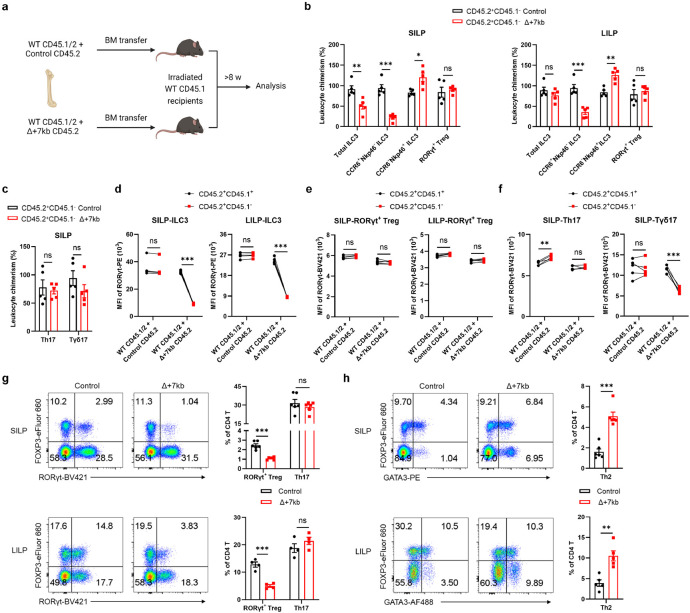
*Rorc(t)* +7kb regulates RORγt^+^ Treg in a cell-extrinsic manner. **a**, Experimental design for the bone marrow (BM) chimeric experiments in **b**-**f**. **b**,**c**, Relative CD45.2^+^CD45.1^−^ leukocyte chimerism normalized to CD45.2^+^CD45.1^−^ splenic B cells (n = 5). **d**-**f**, RORγt mean fluorescence intensity (MFI) of CD45.2^+^CD45.1^+^ and CD45.2^+^CD45.1^−^ RORγt^+^ cells in the SILP and LILP (n = 5). **g**,**h**, Representative flow cytometry plots and frequencies of RORγt^+^ Treg, Th17 and Th2 cells in the SILP and LILP of control (n = 4-6) and Δ+7kb mice (n = 4-6). Data are representative of two (**b**-**f**) or three (**g**,**h**) independent experiments. Data are means ± s.e.m.; ns, not significant; statistics were calculated by unpaired two-sided t-test (**b**,**c**,**g**,**h**) and paired two-sided t-test (**d**-**f**).

Because RORγt is expressed in multiple immune system cell types, the T cell phenotype observed in the Δ+7kb mice could stem from direct or indirect effects. To clarify which cell types are affected by the deletion of the CRE, we established competitive bone marrow (BM) chimeric mice by co-transplanting CD45.1/2 wild-type BM along with either CD45.2 wild-type control or CD45.2 Δ+7kb BM into irradiated CD45.1 wild-type recipients (Extended Data Fig. 4a). We conducted a detailed analysis of donor chimerism across various intestinal immune cell subsets, including RORγt^+^ pTreg, ILC3, Th17, and Tγδ17 cells, normalizing these measurements to splenic B cells as an internal control. As expected, CCR6^+^RORγt^+^ ILC3 derived from the CD45.2 mutant donor were underrepresented, while Nkp46^+^RORγt^+^ ILC3 from the same donor were increased. However, RORγt^+^ pTreg, Th17 and Tγδ17 cells repopulated to similar extents in the mixed chimeras (Extended Data Fig. 4b,c). Moreover, we investigated RORγt expression within RORγt^+^ cell populations, discovering that *Rorc(t)* +7kb intrinsically regulates RORγt expression in ILC3 and Tγδ17, but not in RORγt^+^ pTreg and Th17 cells (Extended Data Fig. 4d-f). Thus, the *Rorc(t)* +7kb mutation does not intrinsically affect T cell differentiation, which is consistent with the observed T cell phenotype being attributed to deficient RORγt-APC function.

## RORγt-APCs required for oral tolerance

**Extended Data Fig. 5 ∣:**
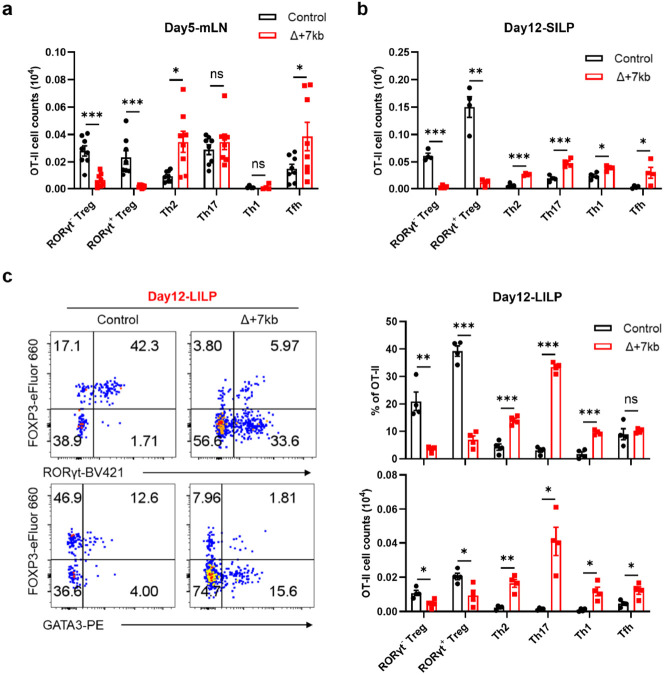
RORγt-APCs are essential for the differentiation of food antigen-specific pTregs. **a**,**b**, Numbers of OT-II pTreg, Th2, Th17, Th1 and Tfh cells in the mLN (**a**) and SILP (**b**) of OVA-treated control and Δ+7kb mice at 5 days and 12 days post-adoptive transfer of naïve OT-II CD4^+^ T cells, respectively. mLN: control mice, n = 8; Δ+7kb mice, n = 8. SILP: control mice, n = 4; Δ+7kb mice, n = 4. **c**, Phenotype of OT-II pTreg, Th2, Th17, Th1 and Tfh cells in the LILP of mice shown in (**b**). Data in **a** are pooled from two independent experiments. Data in **b**,**c** are representative of two (**c**) or three (**b**) independent experiments. Data are means ± s.e.m.; ns, not significant; statistics were calculated by unpaired two-sided t-test.

**Fig. 2 ∣:**
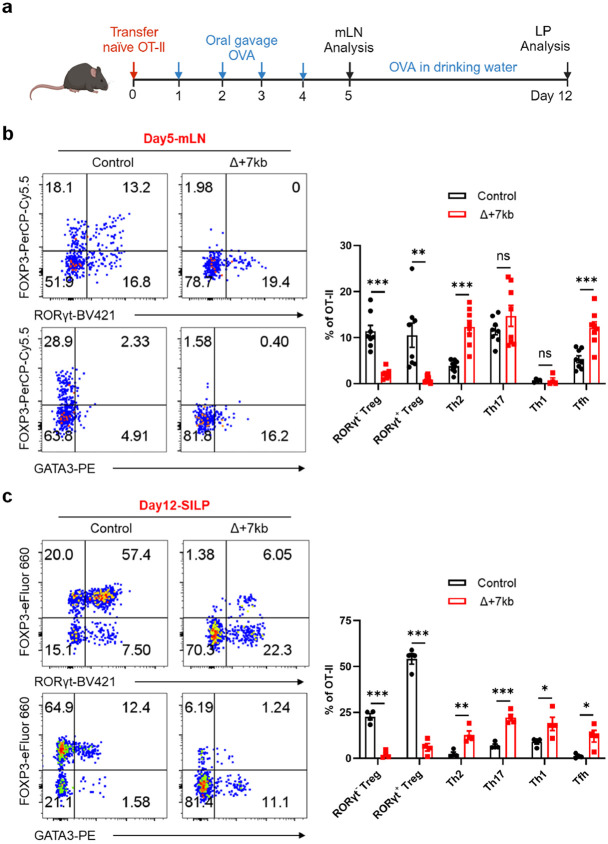
RORγt-APCs promote food antigen-specific pTreg differentiation. **a**, Experimental design. OVA, ovalbumin; mLN, mesenteric lymph nodes; LP, lamina propria. **b**,**c**, Representative flow cytometry plots (left) and frequencies (right) of OT-II pTreg (FOXP3^+^RORγt^+/−^), Th2 (FOXP3^−^GATA3^+^), Th17 (FOXP3^−^GATA3^−^RORγt^+^), Th1 (FOXP3^−^GATA3^−^RORγt^−^T-bet^+^) and Tfh (FOXP3^−^GATA3^−^RORγt^−^T-bet^−^BCL6^+^) cells in the mLN (**b**) and SILP (**c**) of OVA-treated control and Δ+7kb mice at 5 days and 12 days post-adoptive transfer of naïve OT-II CD4^+^ T cells, respectively. mLN: control mice, n = 8; Δ+7kb mice, n = 8. SILP: control mice, n = 4; Δ+7kb mice, n = 4. Data in **b** are pooled from two independent experiments. Data in **c** are representative of three independent experiments. Data are means ± s.e.m.; ns, not significant; statistics were calculated by unpaired two-sided t-test.

When the Δ+7kb mice were examined in the absence of *H. hepaticus* colonization, we observed the expected reduction, compared to wild-type mice, of RORγt^+^ pTregs within both SILP and LILP (Extended Data Fig. 4g). Intriguingly, this decrease did not coincide with an increase in Th17 cell proportions, but was associated with a substantial elevation of intestinal Th2 cell levels (Extended Data Fig. 4g,h). This observation in the mutant mice suggested that tolerogenic APCs induce pTregs specific not only for microbiota, but also for food antigens with potential to induce allergy-related Th2 cells. To test this hypothesis, we transferred naïve ovalbumin (OVA)-specific CD4^+^ T cells from OT-II TCR transgenic mice^29^ into both control and Δ+7kb mice and administered OVA either via gavage or in drinking water (Fig. 2a). Interestingly, OVA-specific OT-II pTregs in wild-type mice consisted of both RORγt^-^ and RORγt^+^ phenotypes (Fig. 2b,c). At five days post-transfer, there were few OT-II pTregs in the mesenteric lymph nodes (mLN) of Δ+7kb mice, and, instead, the OVA-specific T cells displayed Th2 and T follicular helper (Tfh) phenotypes, with no notable changes in Th17 and Th1 profiles (Fig. 2b and Extended Data Fig. 5a). By the twelfth day post-transfer, the reduction in OT-II pTregs persisted in both SILP and LILP of mutant mice, coinciding with variable increases across all OT-II Th cell subsets (Fig. 2c and Extended Data Fig. 5b,c). These results highlight the critical role of RORγt-APCs in promoting food antigen-specific pTreg cell differentiation. A dysfunction of these APCs correlates with intensified effector Th cell responses, although the specific Th cell subset favored depends on the local tissue environment.

**Extended Data Fig. 6 ∣:**
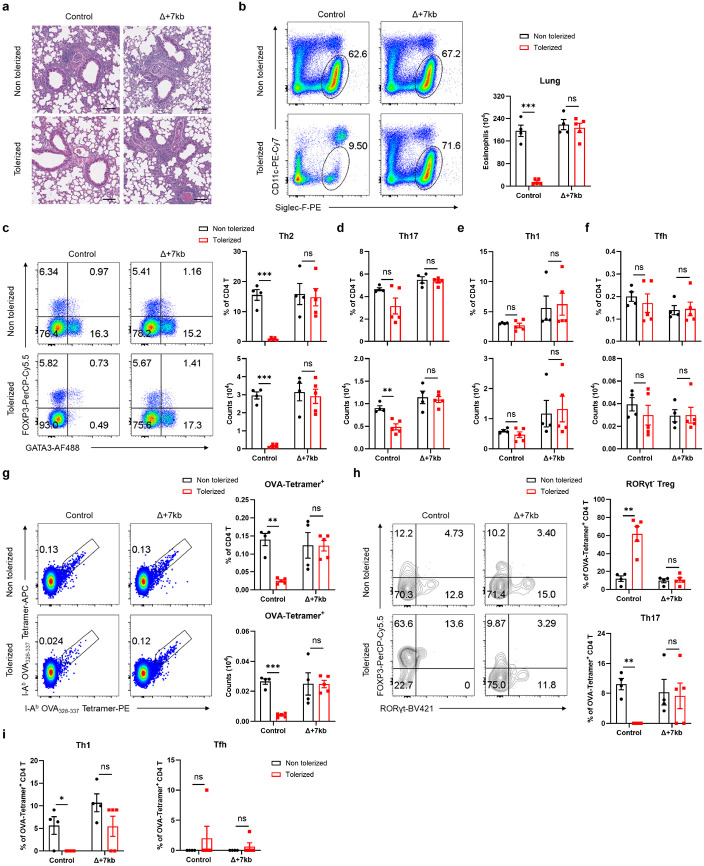
RORγt-APCs are required for establishing oral tolerance against allergic airway responses. **a**, Magnified images of the lung sections in Fig. 3b. Scale bars, 100 μm. **b**-**i**, Eosinophil numbers (**b**), phenotype of total CD4 T cells (**c**-**f**) and OVA:I-A^b^ tetramer^+^ CD4 T cells (**g-i**) in the lung of control and Δ+7kb mice shown in (Fig. 3c-e). Flow cytometry plots in **g,h** were generated by concatenating the samples from each group in FlowJo. Non tolerized control mice, n = 4; tolerized control mice, n = 5; non tolerized Δ+7kb mice, n = 4; tolerized Δ+7kb mice, n = 5. Data are means ± s.e.m.; ns, not significant; statistics were calculated by unpaired two-sided t-test.

**Fig. 3 ∣:**
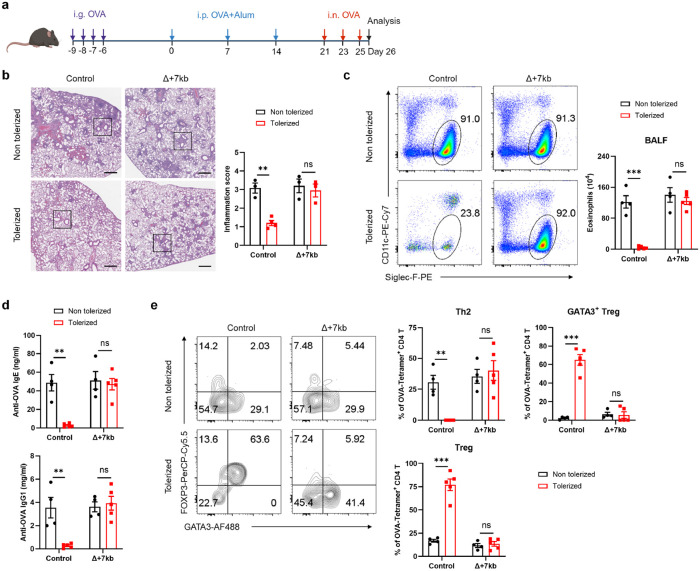
RORγt-APCs are required to develop oral tolerance. **a**, Experimental design. i.g., intragastric; i.p., intraperitoneal; i.n., intranasal. **b**, Hematoxylin and eosin staining and inflammation score of the lung sections in control and Δ+7kb mice. Scale bars, 500 μm. Non tolerized control mice, n = 3; tolerized control mice, n = 4; non tolerized Δ+7kb mice, n = 3; tolerized Δ+7kb mice, n = 3. **c-e**, Eosinophil numbers in the BALF (**c**), OVA-specific IgE and IgG1 in the serum (**d**), phenotype of OVA:I-A^b^ tetramer^+^ CD4 T cells (**e**) in the lung of control and Δ+7kb mice. Flow cytometry plots in **e** were generated by concatenating the samples from each group in FlowJo. Non tolerized control mice, n = 4; tolerized control mice, n = 5; non tolerized Δ+7kb mice, n = 4; tolerized Δ+7kb mice, n = 5. Data are means ± s.e.m.; ns, not significant; statistics were calculated by unpaired two-sided t-test.

Peripheral pTreg cells are pivotal in the induction and maintenance of oral tolerance, a critical mechanism that suppresses diverse immune responses not only in the gastrointestinal tract but also systemically^30,31^. We therefore aimed to explore whether RORγt-dependent APCs are essential for directing food antigen-specific pTregs to mediate oral tolerance. We used an allergic lung response model, in which oral administration of antigen prior to sensitization results in pTreg-mediated inhibition of the inflammatory process. Mice were not pre-treated or were administered OVA intragastrically before being primed with OVA in alum and subsequently exposed to intranasal OVA challenge (Fig. 3a). Wild-type mice that had previously been fed OVA exhibited significant resistance to allergic lung inflammation, demonstrated by lower lung inflammation score, diminished eosinophil numbers in the bronchoalveolar lavage fluid (BALF) and lungs, reduced lung Th2 cells, and decreased levels of serum OVA-specific IgE and IgG1 compared to non-tolerized controls (Fig. 3b-d and Extended Data Fig. 6a-f). However, the same pre-feeding strategy failed to induce oral tolerance in Δ+7kb mice. These knockout mice showed similar increases in lung inflammation score, eosinophils, Th2 cells, and OVA-specific IgE and IgG1 production as non-tolerized Δ+7kb mice (Fig. 3b-d and Extended Data Fig. 6a-f). We then focused on OVA-specific T cell responses in these mice, using OVA:I-A^b^ tetramers to identify those cells. In tolerized wild-type mice, tetramer-positive T cells were significantly fewer compared to non-tolerized animals, and most cells were GATA3^+^FOXP3^+^ Treg cells, with limited RORγt expression (Fig. 3e and Extended Data Fig. 6g,h). In contrast, in both tolerized and non-tolerized Δ+7kb mice there was loss of OVA:I-A^b^ tetramer-binding pTregs in the lung, accompanied by an increase in tetramer-positive Th2, Th17, and Th1 cells, with a predominant increase in Th2 cells (Fig. 3e and Extended Data Fig. 6h,i), consistent with loss of tolerance. These findings indicate that RORγt-APCs are crucial for the development of oral tolerance, highlighting their essential role in regulating immune responses to dietary antigens and preventing allergic responses.

## RORγt regulates Prdm16-expressing APCs

**Fig. 4 ∣:**
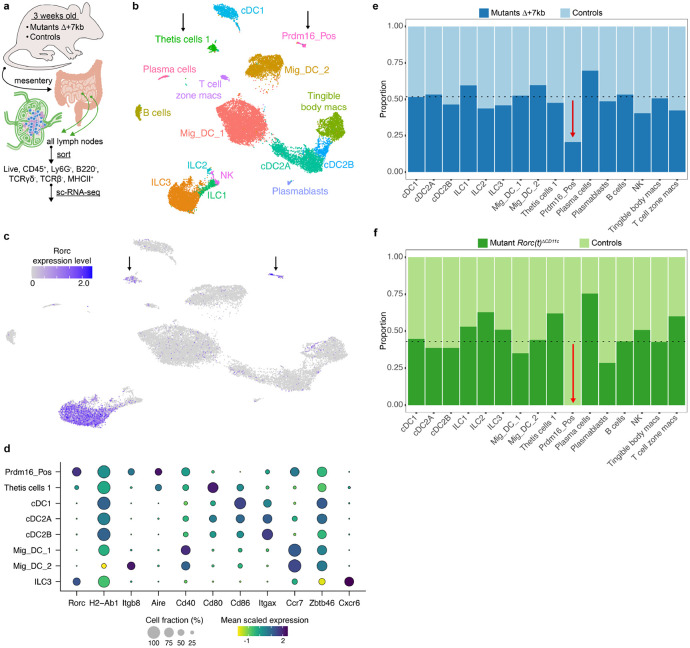
RORγt regulates development of Prdm16-expressing APCs within mLN. **a**, Input scheme for both murine sc-RNA-seq experiments: mLN harvest from mutant and control animals, followed by indicated sorting. **b**,**c**, UMAP of 21,504 resultant transcriptomes from an experiment combining Δ+7kb mutants (n = 7) and controls (n = 7), clustering data together (**b**). Feature plot of *Rorc* expression (**c**). Black arrows label two non-ILC3 clusters positive for *Rorc*. **d**, Dot plot of indicated clusters from **b** examining expression of genes previously ascribed to proposed RORγt-APC subsets. **e**, Stacked bar plots comparing proportion of each cluster in **b**, as derived from Δ+7kb mutant versus control animals. Dotted line at 51.2% indicates total contribution from Δ+7kb mutants. Red arrow indicates 60% loss within Prdm16_Pos cluster of expected Δ+7kb contribution. **f**, Stacked bar plots analogous to experiment **b-e**, but now comparing proportion of cell clusters as derived from *Rorc(t)*^*ΔCD11c*^ mutants versus controls (n = 4 mice, each condition). Dotted line at 43% indicates total contribution from *Rorc(t)*^*ΔCD11c*^ mutants. Red arrow indicates complete loss within Prdm16_Pos cluster of expected mutant contribution.

To better define the RORγt-dependent APCs required for intestinal pTreg cell differentiation, we performed single cell RNA sequencing (sc-RNA-seq) of MHCII-expressing cells from mLN of control and mutant mice, including both Δ+7kb and *Rorc(t)*^Δ*CD11c*^ models. Cells harvested from Δ+7kb mutants and littermate controls at 3 weeks age yielded a total of 21,504 high-quality transcriptomes, comprising 11,150 and 10,354 cells from mutant and control animals, respectively (Fig. 4a,b). We analyzed these data with an unsupervised computation (Methods).

**Extended Data Fig. 7 ∣:**
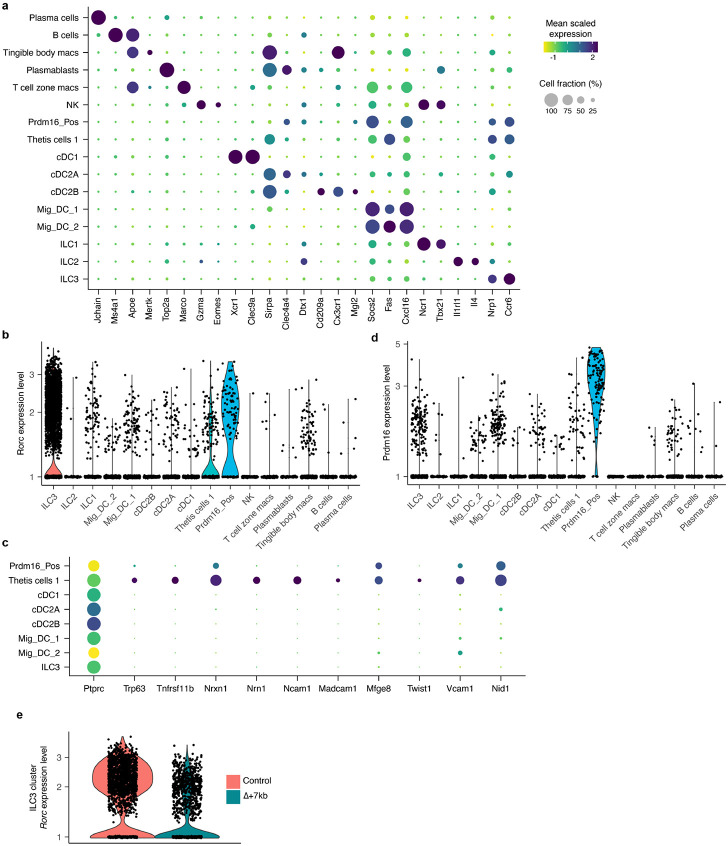
Sequencing annotation and analysis of Δ+7kb mouse model mLN. **a**, Dot plot of all 16 cell types from Δ+7kb mouse model mLN (mutant and control mice combined), demonstrating canonical genes used to annotate each cluster. **b**, Violin plot of *Rorc* expression across all clusters. **c**, Dot plot of select APC clusters, examining genes described for TC I as well as FRC/mTEC cell types. **d**, Violin plot of *Prdm16* expression across all clusters. **e**, Violin plot of *Rorc* expression within the ILC3 cluster, comparing Δ+7kb mutant versus control biological conditions.

Populations of DCs and ILCs and distinct clusters of B cell and macrophage lineages were readily annotated (Fig. 4d, Extended Data Fig. 7a). Juxtaposed ILCs were partitioned based on canonical expression of *Ncr1*/*Tbx21* (Type 1), *Il1rl1*/*Il4* (Type 2), or *Cxcr6/Ccr6/Nrp1* (Type 3), versus a contiguous population of natural killer cells positive for *Eomes* and *Gzma*. cDC1 occupied a distinct cluster positive for *Xcr1* and *Clec9a*. While subsets of cDC2 (all positive for *Sirpa*) were contiguous, they could be separated based on expression of *Clec4a4*/*Cd4*/*Dtx1* (cDC2A) versus *Cd209a*/*Cx3cr1*/*Mgl2* (cDC2B), with all *Tbx21* expression contained within the former, as previously reported^32^. We observed two sizable populations of *Ccr7*^+^ migratory DCs that were denoted Mig_DC_1 and Mig_DC_2, both consistent with a signature known to include *Socs2*, *Fas*, and *Cxcl16*^5,33^.

On querying for *Rorc* positive cells (Fig. 4c and Extended Data Fig. 7b), beyond expected ILC3 there were two additional distinct clusters (arrows, Fig. 4b,c). One population expressed genes consistent with the described TC I subset^6^, including *Aire*, *Trp63*, *Tnfrsf11b*, *Nrxn1*, *Nrn1*, and *Ncam1* (Fig. 4d and Extended Data Fig. 7c). Beyond the TC I gene signature, this cluster also harbored a signature closely associated with the fibroblastic reticular cell (FRC) subset found in mucosal lymph nodes^34^, including *Madcam1*, *Mfge8*, *Twist1*, *Vcam1*, and *Nid1*. Both non-ILC3 *Rorc*^+^ clusters expressed CD45, as well as MHCII at levels comparable to DC subsets. Therefore, the TC I cells are not bona fide stromal cells nor medullary thymic epithelial cells (mTEC), but they harbor prominent FRC and mTEC attributes.

The second *Rorc*^+^ cluster displayed exclusively high expression of *Prdm16*, previously shown to be expressed across the TC subsets^6^ (Extended Data Fig. 7d). The *Prdm16*^+^ cluster was notably higher in *Itgb8*, *Aire*, *Cd40*, and *Ccr7* and lower in *Cd80* and *Cd86* expression when compared to TC I (Fig 4d).

After annotating, we deconvolved each cluster according to its origin from Δ+7kb mutant or control animals. While the contribution from each condition varied minimally around the numeric split of total high-quality transcriptomes captured (Fig. 4e, dotted line), we observed a striking 60% loss of the *Prdm16*^+^ cluster from the expected Δ+7kb contribution. A violin plot examining *Rorc* illustrates a clear decrease of that gene’s expression in the mutant-origin ILC3 population (Extended Data Fig. 7e), yet the overall cluster of ILC3 from mutant mice remained otherwise numerically intact. As there was also no apparent difference in the TC I cluster distribution when comparing control versus Δ+7kb mutant mice, the results suggested that the tolerogenic APCs are contained in the *Prdm16*^+^ population.

**Extended Data Fig. 8 ∣:**
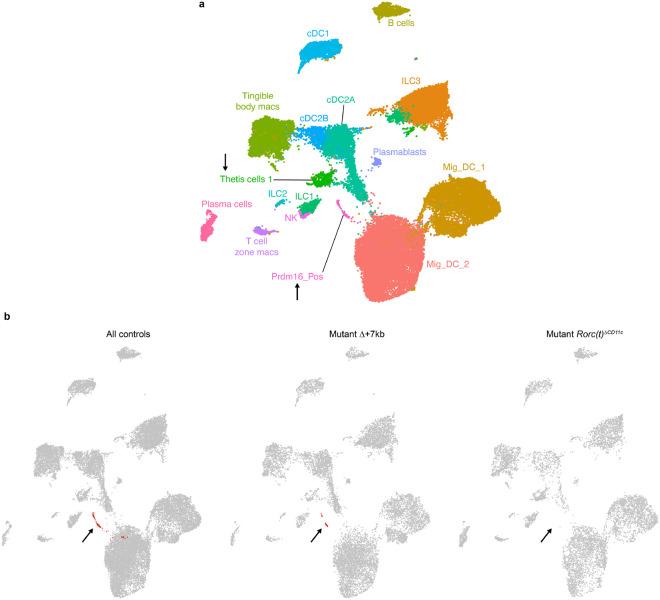
Sequencing of *Rorc(t)*^ΔCD11c^ mouse model and comparison to Δ+7kb model. **a**, Annotated UMAP of 19,719 resultant transcriptomes from a mLN sc-RNA-seq experiment combining *Rorc(t)*^Δ*CD11c*^ mutants and controls, clustering data together. Black arrows label two non-ILC3 clusters positive for *Rorc* expression. **b**, UMAPs concatenating all murine sc-RNA-seq data including mutant Δ+7kb model, mutant *Rorc(t)*^Δ*CD11c*^ model, and all controls (41,923 cells): clustered together and plotted as indicated. Black arrows point to the presence or absence within each biological condition of the Prdm16_Pos cluster shown in red.

**Extended Data Fig. 9 ∣:**
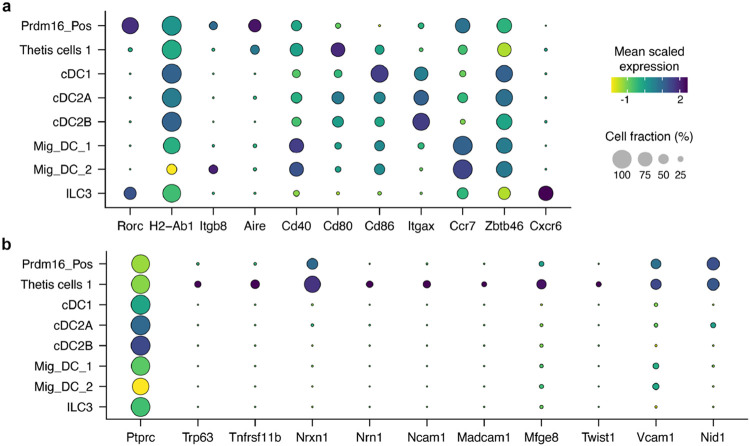
Annotation of *Rorc(t)*^ΔCD11c^ mouse model mLN. **a**, Dot plot of APC clusters examining expression of genes previously ascribed to RORγt-APC subsets. **b**, Dot plot of select APC clusters examining genes described for TC I as well as FRC/mTEC cell types.

Comparison of mLN single cell transcriptomes from *Rorc(t)*^Δ*CD11c*^ (8,474 cells) and littermate control mice (11,245 cells) yielded results congruent with those observed in the Δ+7kb model (Extended Data Fig. 8a and Extended Data Fig. 9a,b). In these mutants, however, there was complete loss of the Prdm16_Pos cluster when we deconvolved biological conditions (Extended Data Fig. 8b), despite a 43% overall contribution of total transcriptomes from *Rorc(t)*^Δ*CD11c*^ mice (Fig. 4f, dotted line). Together, the results are most consistent with a requirement for RORγt expression during development of the *Prdm16*^+^ APCs and suggest that these cells have tolerogenic function in response to both microbiota and food antigens.

**Extended Data Fig. 10 ∣:**
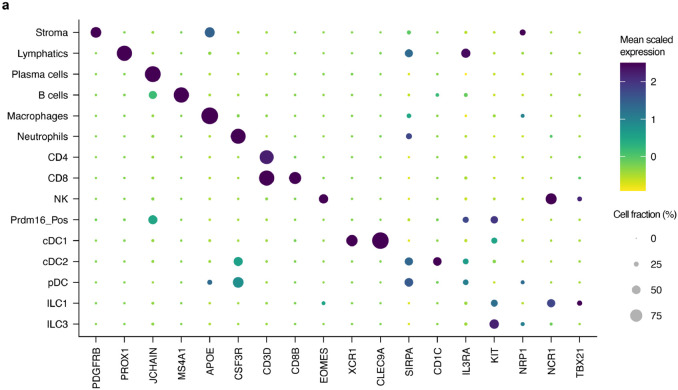
Sequencing annotation of human mLN. **a**, Dot plot of all 15 cell types from human mLN sc-RNA-seq experiment, demonstrating canonical genes used to annotate each cluster.

**Fig. 5 ∣:**
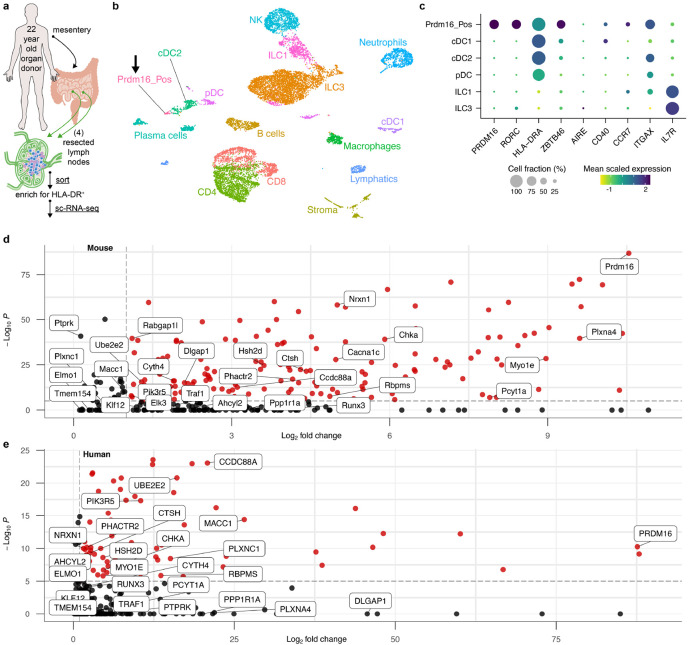
PRDM16-expressing APCs are conserved in human mLN. **a**, Input scheme for human sc-RNA-seq, with four mLN resected and enriched for APCs. **b**, UMAP of 12,928 resultant human mLN transcriptomes. **c**, Dot plot of indicated clusters from **b** examining expression of genes previously ascribed to RORγt-APC subsets. **d**,**e**, Volcano plots of differentially up-regulated genes in the murine (**d**) or human (**e**) Prdm16_Pos cluster compared to the rest of each species’ mLN cell types. A list of 29 genes shared by both species is annotated in each. Red dots indicate differential gene expression meeting *P* > 10^−6^ and Log_2_ fold change > 1.

To determine whether the RORγt-APCs identified in mice are also present in humans, we performed sc-RNA-seq on HLA-DR-enriched cells from freshly isolated mLNs of a 22-year-old trauma patient (Fig. 5a). Following the same unsupervised computation as for the murine experiments, we clustered the resulting single-cell dataset (Fig. 5b). A population of ILC3 was demarcated by expression of *IL7R*, *KIT*, and *NRP1* (Fig. 5c and Extended Fig. 10a). This was juxtaposed by an ILC1 cluster expressing *NCR1* and *TBX21*, as well as NK cells expressing *EOMES*, all consistent with prior literature^35^. A cluster positive for *XCR1* and *CLEC9A* was readily distinguished as cDC1. As compared to murine cDC2, there is less known about human cDC2 that allows for confident subset assignment. Nevertheless, we could identify a *SIRPA*^+^ population overlapping with *CD1C* expression, as would be expected for human cDC2^36^. We annotated a plasmacytoid DC cluster adjacent to this, as these cells were negative for *CD1C* while positive for *IL3RA* (CD123) and included rare cells positive for *TLR7* and *TLR9* expression^37^.

On querying for *RORC* in this human dataset, we observed non-ILC3 expression in a single set of cells clustering at the extremity of the cDC2 annotation (arrow, Fig. 5b,c). Strikingly, this same population exclusively displayed a high *PRDM16* signal. Unlike the murine sc-RNA-seq analysis, this population was not spontaneously demarcated by an unsupervised workflow, so we manually sub-clustered and similarly annotated it as Prdm16_Pos. This human cluster was also positive for *ZBTB46*, *CD40*, *CCR7*, and *ITGAX*, although *AIRE* was not detected in it or any other HLA-DR^+^ mLN population.

Because our results appeared to coincide across datasets, we sought to more closely inspect the non-ILC3 mouse and human cell populations expressing *Rorc* (or *RORC*) for their top differentially upregulated genes as compared to all other cell types within each species’ mLN. This revealed an overlap of 29 genes that were expressed by these cells in both species (Fig. 5d,e). Examining this shared gene list on a volcano plot measuring differential expression within mouse mLN again highlighted *Prdm16* as the most statistically significant gene for this cluster, with a ~1,000-fold change in gene expression compared to all other cell types. Our list also notably included the neural adhesion genes *Nrxn1* (also upregulated in TC I) and *Plxna4*, as well as the transcription factor *Runx3*, which has been described to be required for expression of RORγt in ILC3^38^. Differential expression in the human dataset similarly revealed *PRDM16* to be near top-ranked in terms of statistical significance and fold change gene expression, indicating that this transcription factor is evolutionarily conserved in its co-expression with *RORC* in a presumptive tolerogenic APC.

## Discussion

In maintaining intestinal homeostasis, the gut immune system is tasked with tolerating a complex array of dietary and microbial antigens, largely through the action of peripheral pTregs. This study expands upon the role of RORγt-APCs from their established function in driving microbiota-specific pTreg cell differentiation to facilitating oral tolerance to food antigens. Oral tolerance can be enhanced by oral immunotherapy for food allergies^39,40^ and has been demonstrated effective in animal models to control antigen-specific autoimmune diseases^41,42^. The potential role of RORγt-APCs in modulating autoimmune diseases and transplantation tolerance remains to be elucidated. Our findings indicate that the competency of RORγt-APCs in establishing mucosal tolerance crucially relies on the transcription factor RORγt and a specific CRE within the *Rorc* locus. Because the subset of these cells that also express PRDM16 corresponds to cells found in human mLN^6,43^ and is selectively depleted in the absence of RORγt, we propose that this is the most likely candidate tolerogenic APC. The transcriptional regulatory network in which RORγt participates to influence development and function of these APCs remains to be elucidated. A comprehensive understanding of the key components will likely reveal human genetic variants that can predispose to inflammatory and allergic conditions. An understanding of the ontogeny of the tolerogenic APCs may also provide critical insights into inflammatory diseases and potential therapeutic avenues. Crucially, these tolerogenic APCs possess a remarkable ability to induce pTregs despite their low abundance in the intestinal secondary lymphoid organs. Unraveling the mechanisms behind this potent immunomodulatory effect is essential and will likely require elucidation of the spatial distribution of the APCs and temporal dynamics of differentiation of T cell subsets specific for antigens encountered in the alimentary tract. The unique ability of RORγt-APCs to induce antigen-specific pTreg cells suggests that these APCs could serve as valuable therapeutic targets in multiple immune-related diseases.

## Methods

### Mice

C57BL/6 mice (Jax 000664), CD45.1 mice (B6.SJL-*Ptprc*^*a*^ *Pepc*^*b*^/BoyJ, Jax 002014), CD90.1 mice (B6.PL-*Thy1*^*a*^/CyJ, Jax 000406) and *Cd11c*^cre^ mice (B6.Cg-Tg(Itgax-cre)1-1Reiz/J, Jax 008068) were purchased from the Jackson Laboratories. *Rorc*^fl/fl^, *Rorc(t)*^gfp/gfp^, and Hh7-2tg mice were generated in our laboratory and have been described^15,17,44^. *Il23r*^gfp^ mice^45^ were provided by M. Oukka. OT-II;UBC-GFP mice^29,46^ were provided by S. R. Schwab. Tg (Δ+3kb *Rorc(t)*-mCherry), *Rorc(t)* +6kb^−/−^and *Rorc(t)* +7kb^−/−^ mice were generated as described in ‘Generation of BAC transgenic reporter and CRISPR knockout mice’. Mice were bred and maintained in the Alexandria Center for the Life Sciences animal facility of the New York University School of Medicine, in specific pathogen-free conditions. Sex- and age-matched mice used in all experiments were 8-15 weeks old at the starting point of treatment if not otherwise indicated. All animal procedures were performed in accordance with protocols approved by the Institutional Animal Care and Usage Committee of New York University School of Medicine.

### Generation of BAC transgenic reporter and CRISPR knockout mice

Tg (Δ+3kb *Rorc(t)*-mCherry) mice were generated as previously described^24^. The following primers were used for generating amplicons for GalK recombineering, and screening for correct insertion and later removal of the GalK cassette: Galk Rec +3kb F: CTGCCTCCCACGTGCTAGGATTGTAATATAGAGCATCAGGCCCTGCTCCACCTGTTGACAATT AATCATCGGCA; Galk Rec +3kb R: ACAGACAGATACCATTCCTTGGGCCTGGCTTCCCTCAGTGGTCCTGGCTGTCAGCACTGTCC TGCTCCTT; +3kb HA F: CTGCCTCCCACGTGCTAGGATTGTAATATAGAGCATCAGGCCCTGCTCCA; +3kb HA R: ACAGACAGATACCATTCCTTGGGCCTGGCTTCCCTCAGTGGTCCTGGCTG; +3kb screen F: CTGCCTCCCACGTGCTAGGAT; +3kb screen R: ACAGACAGATACCATTCCTTGGG. The following primers were used for the recombineering that led to scarless deletion of cis-element: deletion template +3kb HA F: CTGCCTCCCACGTGCTAGGATTGTAATATAGAGCATCAGGCCCTGCTCCACAGCCAGGACCA CTGAGGGAAGCCAGGCCCAAGGAATGGTATCTGTCTGT; deletion template +3kb HA R: ACAGACAGATACCATTCCTTGGGCCTGGCTTCCCTCAGTGGTCCTGGCTGTGGAGCAGGGCC TGATGCTCTATATTACAATCCTAGCACGTGGGAGGCAG.

Single guide RNAs (sgRNAs) were designed flanking conserved regions in the +6kb and +7kb region to be deleted using pairs of guides. sgRNAs were cloned into pX458 for use as a PCR template to generate a template for *in vitro* transcription. *In vitro* transcribed sgRNAs and Cas9 mRNA were microinjected into zygotes to generate founder animals that were screened by PCR for deletions. For mice containing expected or interesting deletions, PCR products were TA cloned and Sanger sequenced to determine location of deletion. Founder mice with clean deletions were chosen to breed to C57BL/6 mice to generate lines. The following primers were used to clone sgRNA sequences into BbsI site where lowercase letters represent homology to BbsI: +6/7kb sgRNA-1 F: caccgTTTTCTTTGTGATACCCTTC; +6/7kb sgRNA-1 R: aaacGAAGGGTATCACAAAGAAAAc; +6/7kb sgRNA-2 F: caccGGAGAGACAACTGAAATCGT; +6/7kb sgRNA-2 R: aaacACGATTTCAGTTGTCTCTCC; +6/7kb sgRNA-3 F: caccgTGGACCCAAGTGTTACTGCC; +6/7kb sgRNA-3 R: aaacGGCAGTAACACTTGGGTCCAc.

### Murine antibodies, intracellular staining, and flow cytometry

The following monoclonal antibodies were purchased from Thermo Fisher, BD Biosciences or BioLegend: CD3ε (145-2C11), CD4 (RM4-5), CD11b (M1/70), CD11c (N418), CD25 (PC61.5), CD40 (3/23), CD44 (IM7), CD45 (30-F11), CD45.1 (A20), CD45.2 (104), CD62L (MEL-14), CD90.1 (HIS51), CD90.2 (53-2.1), IL-7R (SB/199), CXCR6 (SA051D1), CCR6 (140706), Nkp46 (29A1.4), MHCII I-A/I-E (M5/114.15.2), Ly6G (1A8), Siglec-F (E50-2440), B220 (RA3-6B2), TCR Vα2 (B20.1), TCRβ (H57-597), TCR Vβ5.1/5.2 (MR9-4), TCR Vβ6 (RR4-7), TCRγδ (GL3), FOXP3 (FJK-16s), RORγt (B2D or Q31-378), GATA3 (TWAJ), T-bet (O4-46), BCL6 (K112-91), and IL-22 (IL22JOP). Anti-mouse CD16/32 (Bio X Cell) was used to block Fc receptors. Live/dead fixable blue (ThermoFisher) was used to exclude dead cells. I-A^b^ OVA_328-337_ tetramers (HAAHAEINEA) were provided by the NIH Tetramer Core Facility.

For labeling OVA-specific T cells, cells were incubated with tetramers for 60 min at 37 °C prior to surface staining. For intracellular staining, cells were stained for surface markers, followed by fixation and permeabilization before intracellular staining according to the manufacturer’s protocol (FOXP3 staining buffer set from Thermo Fisher). For cytokine analysis, cells were stimulated *ex vivo* for 3 h with IL-23 (10 ng/mL; R&D systems) and GolgiStop (BD Biosciences) in complete RPMI-1640 culture medium (RPMI-1640 with 10% FBS, 1% GlutaMAX, 1% penicillin–streptomycin, 10 mM HEPES, and 1 mM sodium pyruvate). Flow cytometric analysis was performed on an LSR II (BD Biosciences) or a Cytek Aurora (Cytek Biosciences) and analyzed using FlowJo software (Tree Star).

### Isolation of lymphocytes

For isolation of cells from lymph nodes and spleens, tissues were mechanically disrupted with the plunger of a 1-ml syringe and passed through 70-μm cell strainers. Bone marrow cells were harvested by flushing out the marrow from cleaned bones using a syringe containing RPMI-1640 wash medium (RPMI-1640 with 3% FBS, 1% GlutaMAX, 1% penicillin–streptomycin, 10 mM HEPES, and 1 mM sodium pyruvate). Red blood cells were lysed with ACK buffer (Thermo Fisher). Cells in bronchoalveolar lavage fluids (BALF) were isolated by flushing the lung with two washes of 0.75 ml PBS via a catheter inserted into a cut made in the trachea.

Lung tissues were cut into small pieces and digested in RPMI-1640 wash medium containing 0.5 mg/ml collagenase D (Sigma) and 0.5 mg/ml DNase I (Sigma) at 37 °C for 45 min with shaking. After removal of Peyer's patches and cecal patches, the intestines were opened longitudinally, cut into 0.5 cm pieces, and washed in PBS twice. Intestines were then incubated with shaking in HBSS medium (without Ca2^+^ and Mg2^+^) containing 3% FBS, 1 mM DTT, 5 mM EDTA, and 10 mM HEPES at 37 °C for 20 min twice. After washing with HBSS medium (without Ca2^+^ and Mg2^+^) containing 3% FBS and 10 mM HEPES, the tissues were then digested in RPMI-1640 wash medium containing 1 mg/ml collagenase D (Sigma), 0.25 mg/ml DNase I (Sigma), and 0.1 U/ml Dispase (Worthington) at 37 °C for 35 min (small intestines) or 55 min (large intestines) with shaking. To isolate leukocytes from the lungs and intestines, the digested tissues were homogenized and passed through 70-μm cell strainers. Mononuclear cells were then collected from the interphase of an 80% and 40% Percoll gradient after a spin at 2,000 rpm for 20 min.

#### *C. rodentium*-mediated colon inflammation

*C. rodentium* strain DBS100 (ATCC51459; American Type Culture Collection) was grown at 37 °C in LB broth. Mice were inoculated with 0.2 ml of a bacterial suspension (2 × 10^9^ CFU) by oral gavage. Mice were followed for the next 14 days to measure body weight change. Fecal pellets were collected and used to measure *C. rodentium* burden with serial dilutions on MacConkey agar plates.

#### *H. hepaticus* culture and oral infection

*H. hepaticus* was provided by J. Fox (MIT). *H. hepaticus* was cultured and administered as previously described^15^. Frozen stock aliquots of *H. hepaticus* were stored in Brucella broth with 20% glycerol and frozen at −80 °C. The bacteria were grown on blood agar plates (TSA with 5% sheep blood, Thermo Fisher). Inoculated plates were placed into a hypoxia chamber (Billups-Rothenberg), and anaerobic gas mixture consisting of 80% nitrogen, 10% hydrogen and 10% carbon dioxide (Airgas) was added to create a micro-aerobic atmosphere, in which the oxygen concentration was 3–5%. The micro-aerobic jars containing bacterial plates were left at 37 °C for 4 days before animal inoculation. For oral infection, *H. hepaticus* was resuspended in Brucella broth by application of a pre-moistened sterile cotton swab applicator tip to the colony surface. 0.2 ml bacterial suspension was administered to each mouse by oral gavage. Mice were inoculated for a second dose after 4 days.

### Adoptive transfer of TCRtg cells

Adoptive transfer of Hh7-2tg CD4^+^ T cells was performed as previously described^15^, with minor modifications. Recipient mice were colonized with *H. hepaticus* by oral gavage 7–8 days before adoptive transfer. Lymph nodes from donor Hh7-2 TCR transgenic mice were collected and mechanically disassociated. Naïve Hh7-2tg CD4^+^ T cells were sorted as CD4^+^TCRβ^+^CD44^low/-^CD62L^+^CD25^−^Vβ6^+^ (Hh7-2tg), on an Aria II (BD Biosciences). Cells were resuspended in PBS on ice and 100,000 cells were then transferred into congenic isotype-labelled recipient mice by retro-orbital injection. Cells from intestines were analyzed 12–14 days after transfer.

Adoptive transfer of OT-II CD4^+^ T cells was performed as previously described^31^, with minor modifications. Lymph nodes from donor OT-II TCR transgenic mice were collected and mechanically dissociated. Naïve OT-II CD4^+^ T cells were sorted as CD4^+^TCRβ^+^CD44^low/−^CD62L^+^CD25^−^Vα2^+^Vβ5.1/5.2^+^ (OT-II), on the Aria II (BD Biosciences). Cells were resuspended in PBS on ice and 100,000 cells were then transferred into congenic recipient mice by retro-orbital injection. Recipient mice received OVA by oral gavage (50 mg; A5378; Sigma) for 4 consecutive days, followed by drinking water containing OVA (2.5 mg/ml) for an additional 7 days after transfer. Cells from mLN were analyzed 5 days after transfer and cells from intestines were analyzed 12 days after transfer.

### Generation of bone marrow chimeric reconstituted mice

To generate chimeric mice, 4- to 5-week-old wild-type CD45.1 mice were irradiated twice with 500 rads per mouse at an interval of 2–5 h (X-RAD 320 X-Ray Irradiator). One day later, mice were reconstituted with bone marrow cells (3–4 × 10^6^) obtained from wild-type CD45.1/2 mice mixed with either CD45.2 Δ+7kb mutant or littermate control bone marrow cells to achieve a ~50:50 chimera. Mice were kept for a week on broad spectrum antibiotics (0.8 mg/ml sulfamethoxazole and 0.16 mg/ml trimethoprim), followed by microbiome reconstitution. After at least 8 weeks, tissues were collected for analysis.

### Induction of allergic airway inflammation

Mice were tolerized by oral gavage with 50 mg of OVA for four consecutive days. Six days after the last gavage, the mice were sensitized three times, one week apart, with intraperitoneal injections of 100 μg of OVA mixed with 1 mg of Alum (1:1; Alhydrogel^®^ adjuvant 2%; vac-alu-50; InvivoGen). Seven days after the final sensitization, the mice were challenged intranasally with 40 μg of OVA every other day, for a total of three challenges. Mice were analyzed 24 h after the last intranasal administration.

### Histology analysis

Lung tissues were perfused and fixed with 10% buffered formalin phosphate, embedded in paraffin, and sectioned. Lung sections were stained with hematoxylin and eosin and scored for histopathology in a blinded fashion as previously described^47^, with minor modifications. Briefly, lung inflammation was assessed by scoring cellular infiltration around the airways and vessels: 0, no infiltrates; 1, few inflammatory cells; 2, a ring of inflammatory cells 1-cell-layer deep; 3, a ring of inflammatory cells 2–3 cells deep; 4, a ring of inflammatory cells 4–5 cells deep; and 5, a ring of inflammatory cells greater than 5 cells deep. The inflammation score for each mouse was calculated as the average of the scores from five lung lobes.

### Enzyme-linked immunosorbent assay

Anti-OVA IgE (439807; BioLegend) and anti-OVA IgG1 (500830; Cayman Chemical) were measured in the serum following the manufacturer’s recommendations.

### Bulk ATAC-seq

Samples were prepared as previously described^24,48^. Briefly, 20-50,000 sort-purified cells were pelleted in a fixed rotor centrifuge at 500 g for 5 minutes, washed once with 50 μL of cold PBS buffer. Spun down again at 500 g for 5 min. Cells were gently pipetted to resuspend the cell pellet in 50 μL of cold lysis buffer (10 mM Tris-HCl, pH7.4, 10 mM NaCl, 3 mM MgCl2, 0.1% IGEPAL CA-630) for 10 minutes. Cells were then spun down immediately at 500 g for 10 min and 4 °C after which the supernatant was discarded and proceeded immediately to the Tn5 transposition reaction. Gently pipette to resuspend nuclei in the transposition reaction mix. Incubate the transposition reaction at 37 °C for 30 min. Immediately following transposition, purify using a Qiagen MinElute Kit. Elute transposed DNA in 10 μL Elution Buffer (10 mM Tris buffer, pH 8.0). The transposed nuclei were then amplified using NEBNext High-fidelity 2X PCR master mix for 5 cycles. In order to reduce GC and size bias in PCR, the PCR reaction is monitored using qPCR to stop amplification prior to saturation using a qPCR side reaction. The additional number of cycles needed for the remaining 45 μL PCR reaction is determined as following: (1) Plot linear Rn vs. Cycle (2) Set 5000 RF threshold (3) Calculate the # of cycle that is corresponded to 1/4 of maximum fluorescent intensity. Purify amplified library using Qiagen PCR Cleanup Kit. Elute the purified library in 20 μL Elution Buffer (10mM Tris Buffer, pH 8). The purified libraries were then run on a high sensitivity Tapestation to determine if proper tagmentation was achieved (band pattern, not too much large untagmented DNA or small overtagmented DNA at the top or bottom of gel. Paired-end 50 bp sequences were generated from samples on an Illumina HiSeq2500. Sequences were mapped to the murine genome (mm10) with bowtie2 (2.2.3), filtered based on mapping score (MAPQ > 30, Samtools (0.1.19)), and duplicates removed (Picard).

### Murine mLN collection, cytometric sorting, and sc-RNA-seq

Live, CD45^+^Lin^-^ APCs were sorted (Fig. 4a) from the mLN of 3-week-old Δ+7kb mutants (n=7) and their littermate controls (n=7), as well as from 17-22 day-old *Rorc(t)*^Δ*CD11c*^ mutants (n=4) and their littermate controls (n=4). Lineage markers comprised TCRβ, TCRγδ, B220, and Ly6G. All libraries were prepared using Chromium Next GEM Single Cell 3-prime Kit v3.1 (10x Genomics), following the vendor’s protocol, and sequenced on an Illumina NovaSeq X+ sequencer.

### Human mLN collection, cytometric sorting, and sc-RNA-seq

We coordinated with a transplant surgery operating room to be notified immediately once a deceased (brain dead) donor was identified for life-saving clinical organ harvest. We procured four mesenteric lymph nodes from a 22-year-old patient maintained on life support, with minimal time from surgical resection to digestion of tissue at the benchside, resulting in >92% viable cells. The donor was free of chronic diseases and cancer, and negative for hepatitis B, hepatitis C, and HIV. This study does not qualify as human subjects research, as confirmed by NYU Langone Institutional Review Board, because tissues were obtained from a de-identified deceased individual.

The nodes were transported in Belzer UW Cold Storage Solution, and resected for excess adipose. Digestion buffer comprised RPMI media with 0.2mg/mL Liberase Thermolysin Medium and 2.5mg/mL collagenase D. Each node was pierced with a 31G syringe, injected with digestion buffer until plump, and submerged in the same for 30 minutes at 37 degrees under agitation. Placing onto 100 micron filters, nodes were smashed open with syringe plungers, washing with excess RPMI to maximally recover cells. The suspension was stained with Live/dead eFluor780 (ThermoFisher), CD19 and CD3 (Biolegend HIB19 and UCHT1), and HLA-DR (BD Biosciences L243). Cells were initially gated to enrich viable non-lymphocyte singlets (Fig. 5a). Not knowing MHCII expression levels within human nodes a priori, we chose to sort a wide range of HLA-DR^+^ stained cells, omitting only the very bottom quartile. Sorting 61,232 cells ultimately allowed loading of 26,000 across 2 lanes of a 10X microfluidics chip, followed by the same library preparation and sequencing as for murine experiments.

### Computational analysis

RNA-sequencing data were aligned to reference genomes mm10-2020-A (mouse) or GRCh38-2020-A (human) and counted by Cell Ranger (v7.1.0) with default quality control parameters. Each dataset was filtered, removing cells with fewer than 500 detected genes, those with an aberrantly high number of genes (more than 10,000), and those with a high percentage of mitochondrial genes (more than 5%). We computed cell cycle scores for known S-phase and G2/M-phase marker genes, regressing out these variables, along with mitochondrial and ribosomal protein genes.

We employed a completely unsupervised computational workflow that analyzed all cells in aggregate, utilizing the most recent methods^49^ within Seurat version 5.1 (including *sctransform* function) for normalization of gene expression, anchor-based integration (based on 3000 features), and shared nearest neighbor cluster identification. We performed dimensional reduction using a principal component analysis that retained 50 dimensions, and clustering with the Leiden algorithm set to resolution = 1.0. This matched together cells with shared biological states across mutant and control animals, ensuring the final clusters were not driven by effects within either condition. The *Rorc(t)*^Δ*CD11c*^ murine sc-RNA-seq dataset was appended to the Δ+7kb dataset, also clustered in an unsupervised manner, and then analyzed for the same populations as before. The human sc-RNA-seq dataset also underwent a predominantly unsupervised pipeline, but the Prdm16_Pos population was subsequently manually sub-clustered from a juxtaposed cDC2 population.

### Statistical analysis

Unpaired two-sided t-test and paired two-sided t-test were performed to compare the results using GraphPad Prism, Version 10 (GraphPad Software). No samples were excluded from analysis. We treated less than 0.05 of P value as significant differences. *P < 0.05, **P < 0.01, and ***P < 0.001. Details regarding number of replicates and representative data can be found in figure legends.

## Extended Data
